# Intermittent Androgen Suppression: Estimating Parameters for Individual Patients Based on Initial PSA Data in Response to Androgen Deprivation Therapy

**DOI:** 10.1371/journal.pone.0130372

**Published:** 2015-06-24

**Authors:** Yoshito Hirata, Kai Morino, Koichiro Akakura, Celestia S. Higano, Nicholas Bruchovsky, Teresa Gambol, Susan Hall, Gouhei Tanaka, Kazuyuki Aihara

**Affiliations:** 1 Institute of Industrial Science, The University of Tokyo, Meguro-ku, Tokyo 153-8505, Japan; 2 Graduate School of Information Science and Technology, The University of Tokyo, Tokyo, Japan; 3 Department of Urology, JCHO Tokyo Shinjuku Medical Center, Japan Community Health Care Organization Tokyo, Japan; 4 Department of Medicine, University of Washington and Fred Hutchinson Cancer Research Center, Seattle, Washington, USA; 5 Vancouver Prostate Centre, Vancouver, BC, Canada; 6 Graduate School of Engineering, The University of Tokyo, Tokyo, Japan; University of California-Irvine, UNITED STATES

## Abstract

When a physician decides on a treatment and its schedule for a specific patient, information gained from prior patients and experience in the past is taken into account. A more objective way to make such treatment decisions based on actual data would be useful to the clinician. Although there are many mathematical models proposed for various diseases, so far there is no mathematical method that accomplishes optimization of the treatment schedule using the information gained from past patients or “rapid learning” technology. In an attempt to use this approach, we integrate the information gained from patients previously treated with intermittent androgen suppression (IAS) with that from a current patient by first fitting the time courses of clinical data observed from the previously treated patients, then constructing the prior information of the parameter values of the mathematical model, and finally, maximizing the posterior probability for the parameters of the current patient using the prior information. Although we used data from prostate cancer patients, the proposed method is general, and thus can be applied to other diseases once an appropriate mathematical model is established for that disease.

## Introduction

Mathematical models for diseases [1–20] are valuable for helping medical doctors optimize therapy based on characteristics of the individual patient’s tumor behavior. When we apply a mathematical model to a series of data points obtained over time for an individual patient, the duration of time over which these data points are obtained should be as short as possible for the model to have practical implications.

In clinical practice, a medical doctor often chooses a treatment option for a current patient based on prior limited observations and experiences gained from prior patients. Use of a mathematical model based on data obtained from many prior patients as well as use of computational technology would permit more objective decision making for the individual patient. However, such a method has not been proposed yet as far as we are aware of.

In this paper, we apply a mathematical model derived from data of men treated with intermittent androgen suppression (IAS) [21–23] to current patients to assess how the model performs. First, we construct the prior information for the parameters of the mathematical model by fitting data from patients treated over longer intervals. Then, we fit the data from a shorter time course obtained from the current patient using the Bayesian formula [24].

## Materials and Methods

### Mathematical model of disease

We assume that a mathematical model of disease is given by a dynamical system. Namely, suppose that the state vector $\vec{x}\in R^{r}$ represents the internal state of the disease, where *r* is the number of state variables. In addition, assume that the dynamical system $f_{\vec{q}}^{m}:R^{r}\to R^{r}$ is given by

$$\begin{array}{c} \frac{d\vec{x}}{dt}=f_{\vec{q}}^{m}\left(\vec{x}\right). \end{array}$$ (1)

Here $\vec{q}$ is a set of parameters for a patient of the disease, and *m* shows whether a treatment is on (*m* = 1) or off (*m* = 0). We assume that given a set of initial conditions $\vec{x}(0)$, a set of parameters $\vec{q}$, and a series of times for starting and stopping the treatment, then the solution $\vec{x}(t)$ exists uniquely. Although we cannot observe $\vec{x}(t)$ directly, we can have some measurement $g(\vec{x}(t))$ through an observation function *g* : *R* ^*r*^ → *R*. Typically, $g(\vec{x}(t))$ corresponds to measurement of some biomarker. To optimize a series of times for deciding when we stop and/or resume the treatment in the future, it is important to decide what are a set of initial conditions $\vec{x}(0)$ and parameters $\vec{q}$. We denote a set of $\left(\vec{x}(0),\vec{q}\right)$ by $\vec{p}$, which is called a set of combined parameters. Our problem is how to determine $\vec{p}$ given a series of times for starting and/or stopping the treatment, and a short series of observations $\vec{o}=\{o_{k}\in R|k=1,2,\ldots ,K\}$ corresponding to the values $\left\{{\hat{o}}_{k}=g(\vec{x}(t_{k}))\right\}$ for the generated orbit at discrete days {*t*_*k*_∣*t*_*k*_ ≥ 0, *k* = 1, 2, …, *K*}.

### Bayesian formula

We employed the Bayesian formula [24] to estimate a set of combined parameter values for the current patient by using the prior information obtained from the past other patients. The Bayesian formula enables us to estimate the probability for the combined parameter values given a series of observations from the prior probability for the combined parameter values and the probability for the series of observations given the combined parameter values. Initially, we obtained the prior probability for the combined parameter values by fitting entire series of observations for the past other patients. Then, for each patient for whom we now want to estimate their set of combined parameters, we employ a dynamical model of disease to obtain the probability for observations given a set of combined parameter values. Then, we maximize the probability for the combined parameter values, and hence the likelihood for the generated orbit given the series of observations to obtain the combined parameter values.

Mathematically we use the Bayesian formula [24] to describe the posterior probability $P(\vec{p}|\vec{o})$ of combined parameters $\vec{p}$ given observation $\vec{o}$ of biomarker time series by combining the conditional probability $P(\vec{o}|\vec{p})$ of observation $\vec{o}$ given the combined parameters $\vec{p}$ with the prior probability $P(\vec{p})$ for the combined parameters [24] as follows:

$$\begin{array}{c} P\left(\vec{p}|\vec{o}\right)\propto P\left(\vec{o}|\vec{p}\right)P\left(\vec{p}\right). \end{array}$$ (2)

By taking the logarithm, we obtain the following equation with a constant:

$$\begin{array}{c} \log P\left(\vec{p}|\vec{o}\right)=\log P\left(\vec{o}|\vec{p}\right)+\log P\left(\vec{p}\right)+c. \end{array}$$ (3)

In addition, suppose that there are constraints $C_{a}(\vec{p})\geq 0$ for *a* = 1, …, *A* and $D_{b}(\vec{p})=0$ for *b* = 1, …, *B* that realize physiological appropriateness for the disease. Using

$$\begin{array}{c} h\left(e\right)=\{\begin{array}{cc} 0, & e\geq 0,\\ 10000(1-e), & e<0, \end{array} \end{array}$$ (4)

we combine these constraints in $Q(\vec{p})$ using the penalty method [25] as

$$\begin{array}{c} Q\left(\vec{p}\right)=\sum_{a=1}^{A}h\left(C_{a}\left(\vec{p}\right)\right)+\sum_{b=1}^{B}\{h\left(D_{b}\left(\vec{p}\right)\right)+h\left(-D_{b}\left(\vec{p}\right)\right)\}. \end{array}$$ (5)

Observe that $Q(\vec{p})$ is non-negative and attains $Q(\vec{p})=0$ when all the constraints are fulfilled. See Eq 22 for a more concrete example of $Q(\vec{p})$. Although we used the penalty method to implement these constraints, readers might use the method of Lagrangian alternatively.

By flipping the sign of the right-side of Eq 3, we minimize

$$\begin{array}{c} -\log P\left(\vec{o}|\vec{p}\right)-\log P\left(\vec{p}\right)+Q\left(\vec{p}\right) \end{array}$$ (6)

to obtain a set of the combined parameters $\vec{p}$. Namely, the optimal and personalized combined parameters $\vec{p}$ can be written as

$$\begin{array}{c} \hat{p}=\underset{\vec{p}}{\text{arg}\;\text{min}}\{-\log P\left(\vec{o}|\vec{p}\right)-\log P\left(\vec{p}\right)+Q\left(\vec{p}\right)\}. \end{array}$$ (7)

Through the minimization, we can obtain a set of combined parameters that realizes the physiological appropriateness discussed above, as well as achieving that the corresponding generated orbit $\left\{\vec{x}(t)\right\}$ is the most likely given the observed dataset $\vec{o}$.

There are many possible ways to prepare the prior distribution $P(\vec{p})$. In this paper, we fit time series of biomarker for prior patients, obtain the mean $\bar{p}$ and the co-variance matrix Σ, and approximate $P(\vec{p})$, for our first step, by a multivariate Gaussian distribution [24] as

$$\begin{array}{c} P\left(\vec{p}\right)=\frac{1}{\sqrt{{\left(2\pi \right)}^{n}\left|\Sigma \right|}}\exp\{-\frac{{\left(\vec{p}-\bar{p}\right)}^{T}\Sigma^{-1}\left(\vec{p}-\bar{p}\right)}{2}\}, \end{array}$$ (8)

where *n* is the number of the combined parameters. Let *o*_*i*_ and ${\hat{o}}_{i}$ be the actual value for the *i*th observation and its estimation by the mathematical model. Because the prediction error $-\text{log}\;P(\vec{o}|\vec{p})$ can be written as

$$\begin{array}{c} \frac{1}{2\sigma^{2}}\sum_{k}{\left(o_{k}-{\hat{o}}_{k}\right)}^{2} \end{array}$$ (9)

plus a constant value if we assume that the observational noise is Gaussian with mean 0 and standard deviation *σ*, Eq 7 can be rewritten as

$$\begin{array}{c} \hat{p}=\underset{\vec{p}}{\text{arg}\;\text{min}}\left\{\frac{1}{2\sigma^{2}}\sum_{k}{\left(o_{k}-{\hat{o}}_{k}\right)}^{2}+\frac{1}{2}{\left(\vec{p}-\bar{p}\right)}^{T}\Sigma^{-1}\left(\vec{p}-\bar{p}\right)+Q\left(\vec{p}\right)\right\}. \end{array}$$ (10)

We solve this minimization problem using differential evolution [25], a heuristic method for minimization. Differential evolution is one of variants of genetic algorithms [25].

### Intermittent androgen suppression as an example

In this paper, we use a mathematical model derived from prostate cancer patients treated with IAS [15] as an example. Prostate cancer is dependent on testosterone for growth and therefore, suppression of testosterone production (usually accomplished with a GnRH analog) is the mainstay of therapy for metastatic prostate cancer. It is also used in men who have only biochemical evidence of recurrent prostate cancer after either surgery or radiation therapy. While androgen suppression is usually very effective early in the disease, eventually the cancer cells acquire the ability to survive even when serum androgen levels are in the castrate range. This disease state is called castration resistant prostate cancer (CRPC).

Intermittent androgen suppression delayed time to CRPC compared to continuous androgen suppression in animal models and has been studied in numerous clinical trials in an effort to delay time to CRPC as well as to improve quality of life [26, 27]. In clinical trials of IAS, androgen suppression is generally stopped after 9–12 months of treatment and is resumed when PSA increases above a pre-specified threshold.

Although there are many mathematical models [9–11, 13–20] proposed until now, we used the model of Ref. [15] because there are two published papers [28, 29] that compared the model of Ref. [15] with that of Ref. [19], concluding that the model of Ref. [15] is relatively better, especially in quality on whether relapse can be reproduced or not (the model of Ref. [19] could not reproduce the relapse of prostate cancer under the assumption of continuous androgen suppression with the combined parameters obtained after fitting the real data), while Ref. [28] showed that the model of Ref. [15] outperformed the model of Ref. [19] when they were compared in the mean square prediction errors although their difference in predictability is not yet significant. We have not yet compared the model of Ref. [15] with other models such as the one in Ref. [18] because the model of Ref. [18] contains many parameters and might need longer data to fit them while the lengths of our datasets are limited.

In the mathematical model of Hirata et al. [15], multiple pathways of castration resistance are summarized into two categories: reversible (epigenetic) and irreversible (genetic). In the model, there are three variables: the first one *x*_1_ for androgen dependent cancer (AD) cells, and the other two *x*_2_ and *x*_3_, androgen independent (castration resistant) cancer (AI) cells with reversible and irreversible changes, respectively. When we apply hormone therapy, AD cells whose population is expressed by *x*_1_ may change to the AI cells whose populations are expressed by *x*_2_ and *x*_3_. In addition, the AI cells in *x*_2_ can change to the AI cells in *x*_3_. If we stop the hormone therapy, the AI cells in *x*_2_ may change back to *x*_1_, AD cells. However, the AI cells in *x*_3_ would not change back to *x*_1_ because of genetic mutation. Therefore, the mathematical model can be described as

$$\begin{array}{c} \frac{d}{dt}(\begin{array}{c} x_{1}\\ x_{2}\\ x_{3} \end{array})=(\begin{array}{ccc} w_{1,1}^{1} & 0 & 0\\ w_{2,1}^{1} & w_{2,2}^{1} & 0\\ w_{3,1}^{1} & w_{3,2}^{1} & w_{3,3}^{1} \end{array})(\begin{array}{c} x_{1}\\ x_{2}\\ x_{3} \end{array}) \end{array}$$ (11)

for the on-treatment periods and

$$\begin{array}{c} \frac{d}{dt}(\begin{array}{c} x_{1}\\ x_{2}\\ x_{3} \end{array})=(\begin{array}{ccc} w_{1,1}^{0} & w_{1,2}^{0} & 0\\ 0 & w_{2,2}^{0} & 0\\ 0 & 0 & w_{3,3}^{0} \end{array})(\begin{array}{c} x_{1}\\ x_{2}\\ x_{3} \end{array}) \end{array}$$ (12)

for the off-treatment periods. The parameter $w_{i,j}^{m}$ means the contribution of *x*_*j*_ to the growth of *x*_*i*_ for the treatment period labeled by *m* (*m* = 1 during the on-treatment periods and *m* = 0 during the off-treatment periods). We assume that we can observe the PSA level which is given by *x*_1_ + *x*_2_ + *x*_3_ for simplicity.

**Fig 1:**
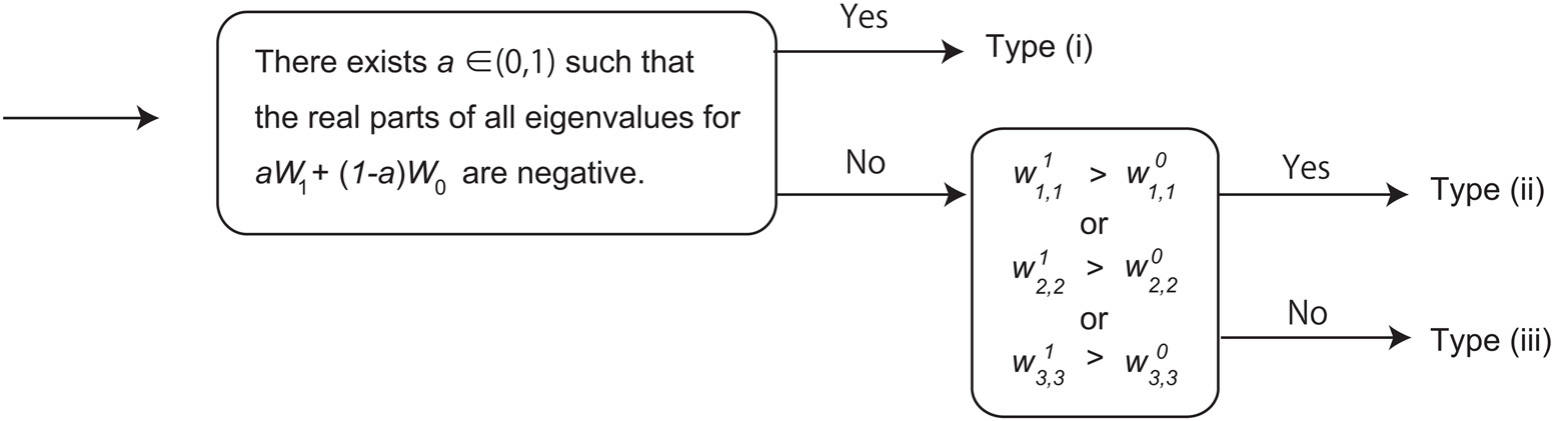
Classification of patients for intermittent androgen suppression using the mathematical model of Ref. [15]. These criteria used here were originally proposed in Ref. [30].

Patients are classified into 3 types [15, 30] (see Fig 1): Type (i) is for patients whose relapse, namely the increase in PSA, can be prevented by IAS; Type (ii) is for patients whose relapse cannot be prevented eventually but can be delayed by IAS later than the continuous androgen suppression (CAS); Type (iii) is for patients for whom CAS is more desirable than IAS in a long run. In this paper, we follow the criteria proposed in Ref. [30].

When we fit the datasets for Eqs 11 and 12, we use the Euler approximation and describe the dynamics with difference equations as

$$\begin{array}{c} \left(\begin{array}{c} x_{1}\left(t+\Delta t\right)\\ x_{2}\left(t+\Delta t\right)\\ x_{3}\left(t+\Delta t\right) \end{array})=(\begin{array}{ccc} d_{1,1}^{1} & 0 & 0\\ d_{2,1}^{1} & d_{2,2}^{1} & 0\\ d_{3,1}^{1} & d_{3,2}^{1} & d_{3,3}^{1} \end{array})(\begin{array}{c} x_{1}\left(t\right)\\ x_{2}\left(t\right)\\ x_{3}\left(t\right) \end{array}\right) \end{array}$$ (13)

for the on-treatment periods and

$$\begin{array}{c} \left(\begin{array}{c} x_{1}\left(t+\Delta t\right)\\ x_{2}\left(t+\Delta t\right)\\ x_{3}\left(t+\Delta t\right) \end{array})=(\begin{array}{ccc} d_{1,1}^{0} & d_{1,2}^{0} & 0\\ 0 & d_{2,2}^{0} & 0\\ 0 & 0 & d_{3,3}^{0} \end{array})(\begin{array}{c} x_{1}\left(t\right)\\ x_{2}\left(t\right)\\ x_{3}\left(t\right) \end{array}\right) \end{array}$$ (14)

for the off-treatment periods, where $d_{i,i}^{m}=(1+w_{i,i}^{m}\Delta t)$, $d_{i,j}^{m}=w_{i,j}^{m}\Delta t$ for *i* ≠ *j*, and we set Δ*t* = 1 (day). In addition, we enforce some constraints to realize the biological appropriateness during fitting the datasets for the mathematical model [15]. These constraints include the non-negativity for the parameters and initial conditions, the bounds for changes in the cell type within a day, and the possibility to relapse if we continue the hormone therapy by CAS. We use the penalty method [25] to enforce these constraints. These constraints can be written as

$$\begin{array}{c} d_{i,j}^{m}\geq 0 \end{array}$$ (15)

for each *i*, *j*, *m*,

$$\begin{array}{c} x_{i}\left(0\right)\geq 0 \end{array}$$ (16)

for each *i*,

$$\begin{array}{c} 0.8\leq d_{i,i}^{m}\leq 1.2 \end{array}$$ (17)

for each *i*, *m*,

$$\begin{array}{c} d_{2,1}^{1}\leq 0.1,d_{3,1}^{1}\leq 0.1,d_{3,2}^{1}\leq 0.1,d_{1,2}^{0}\leq 0.1, \end{array}$$ (18)

$$\begin{array}{c} 0.8\leq \sum_{i=1}^{3}d_{i,1}^{1}\leq 1.2,0.8\leq \sum_{i=2}^{3}d_{i,2}^{1}\leq 1.2,0.8\leq d_{i,2}^{0}\leq 1.2, \end{array}$$ (19)

$$\begin{array}{c} d_{3,3}^{1}\geq 1, \end{array}$$ (20)

and

$$\begin{array}{c} \sum_{i}{\bar{x}}_{i}\left(360\right)\leq 2,\sum_{i}{\bar{x}}_{i}\left(360\times 5\right)\geq 10, \end{array}$$ (21)

where ${\bar{x}}_{i}(t)$ is the *i*th component of the solution at time *t* assuming the continuous androgen suppression. Thus, using *h*(*e*) in Eq 4, the non-negative fucntion $Q(\vec{p})$ in this model can be written as

$$\begin{array}{c} \begin{array}{ccc} Q(\vec{p}) & = & \Sigma_{i,j,m}h\left(d_{i,j}^{m}\right)+\Sigma_{i}h\left(x_{i}\left(0\right)\right)+\Sigma_{i,m}\left(h\left(d_{i,i}^{m}-0.8\right)+h\left(1.2-d_{i,i}^{m}\right)\right)\\ & & +h\left(0.1-d_{2,1}^{1}\right)+h\left(0.1-d_{3,1}^{1}\right)+h\left(0.1-d_{3,2}^{1}\right)+h\left(0.1-d_{1,2}^{0}\right)\\ & & +h\left(\Sigma_{i=1}^{3}d_{i,1}^{1}-0.8\right)+h\left(1.2-\Sigma_{i=1}^{3}d_{i,1}^{1}\right)+h\left(\Sigma_{i=2}^{3}d_{i,2}^{1}-0.8\right)+h\left(1.2-\Sigma_{i=2}^{3}d_{i,2}^{1}\right)\\ & & +h\left(\Sigma_{i=1}^{2}d_{i,2}^{0}-0.8\right)+h\left(1.2-\Sigma_{i=1}^{2}d_{i,2}^{0}\right)+h\left(d_{3,3}^{1}-1\right)\\ & & +h\left(2-\Sigma_{i}{\bar{x}}_{i}\left(360\right)\right)+h\left(\Sigma_{i}{\bar{x}}_{i}\left(360\times 5\right)-10\right). \end{array} \end{array}$$ (22)

Therefore, here $\vec{p}$ consists of parameters $d_{i,j}^{m}$ and initial conditions *x*_*i*_(0) in this example.

We used differential evolution [25] for solving the minimization problem of Eq 7, and estimating the optimal set of the combined parameters $\hat{p}$. See the supporting information for our program implementing the proposed method in C. We can clearly see whether or not all the constraints are satisfied because the cost function becomes smaller than 10000 if all the constraints are satisfied.

### Data

We used three datasets in this study. The first dataset is that obtained from the Canadian Phase II clinical trial [22, 23] of IAS (There are neither registry name nor registration number for this trial because it is too old). The Health Clinical Research Ethics Review Boards of each participating center approved this study and all patients signed written informed consent. This study was previously analyzed in Refs. [15, 16, 30, 31]. We divided 72 patients into 2 groups. The combined parameters of the model for the first 36 patients were fitted using the whole datasets in the way described in Ref. [15]. Then, we obtained the mean vector and the covariance matrix for their combined parameters to construct the prior information. We used the remaining 36 patients for testing the proposed method.

The second and the third datasets are from the studies performed in Japan and the United States. The ethics committees of JCHO Tokyo Shinjuku Medical Center and University of Tokyo approved the Japanese study of 26 patients. In the Japanese study, all the patients provided informed consent orally. The ethics committee of JCHO Tokyo Shinjuku Medical Center approved that the oral consent is sufficient because the study was part of usual clinical practice, retrospective, and did not intervene their treatments. The medical doctor for each patient documented the patient’s oral consent by writing down his agreement on his medical record. This Japanese study does not have the registry name or the registration number because it is not a clinical trial. This dataset was previously analyzed in Ref. [30]. The University of Washington Institutional Review Board approved the American study, an on-going Phase II study of IAS [32, 33] that included 79 patients. The NCI number for this American trial is NCT00223665. In the American study, all the patients provided written informed consent. This American study was also analyzed previously in Ref. [30]. These datasets comprised of a total of 141 patients were also used for testing the proposed method. We set $\sigma =1/\sqrt{2}$ because the mean and the standard deviation for the residuals per measurement for the first 36 patients using the fitting method of Ref. [15] were 0.64±0.29, including $1/\sqrt{2}$ within this error bar.

## Results

We compared the classifications obtained from fitting the whole data by the method of Ref. [15] with those obtained from fitting the whole data by the proposed method. These two groups of the classifications are shown in Tables 1 and 2, respectively. Although the method of Ref. [15] lost the correlation between the classifications by the mathematical model and those by the medical doctors when we restricted the datasets to the second half of patients (see Table 1, especially the p-value of 0.13), the proposed method kept the correlation by using the prior distribution (see Table 2, which achieved the smaller p-value less than 0.001 due to the fact that most patients without relapse were classified into Type (i), while most patients with metastasis and androgen independence were classified into Type (ii)). In addition, Table 3 shows that the classifications by the proposed method and the whole dataset retain the typical characteristics of the classifications by the method of Ref. [15] because more than the half of the patients concentrated on the diagonal elements within this table. Therefore, the proposed method seems to work well.

**Table 1:** Results obtained by the original method used in Ref. [15]. MD and MM [15] correspond to classifications by medical doctors and those by mathematical models using the fitting method of Ref. [15], respectively (the p-value obtained by Fisher’s exact test implemented in R (we applied the same method for obtaining the p-values in the other tables): 0.13). For example, there were 35 patients who were classified to “Without relapse” by medical doctors and were classified to “Type (i)” by the mathematical models using the original method of Ref. [15].

	MM [15]			
MD	Type(i)	Type(ii)	Type(iii)	Total
Without relapse	35	42	4	81
Metastasis	7	13	1	21
Androgen independence	9	22	6	37
Total	51	77	11	139

**Table 2:** Results obtained by the proposed method. MD and MMWD correspond to classifications by medical doctors and those by mathematical models with the whole data, respectively (the p-value < 0.001).

	MMWD			
MD	Type(i)	Type(ii)	Type(iii)	Total
Without relapse	61	14	6	81
Metastasis	9	9	3	21
Androgen independence	12	18	7	37
Total	82	41	16	139

**Table 3:** Comparison between the classifications obtained by the proposed method with the whole data (MMWD) using the classifications by mathematical models using the fitting method of Ref. [15] (MM[15]) (p-value < 0.001).

	MM [15]			
MMWD	Type (i)	Type (ii)	Type (iii)	Total
Type (i)	40	40	3	83
Type (ii)	9	30	3	42
Type (iii)	4	7	5	16
Total	53	77	11	141

**Fig 2:**
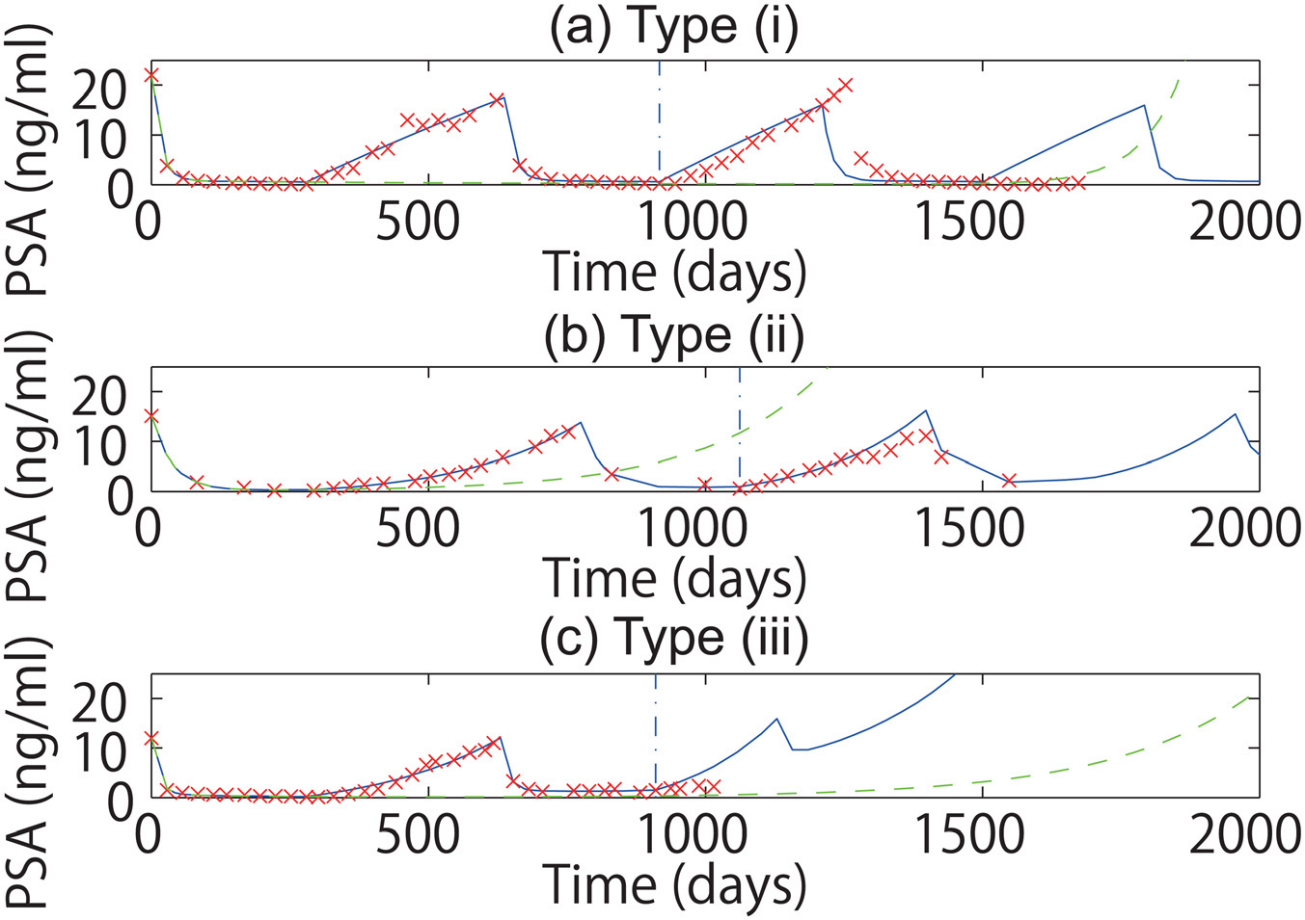
Fitting and prediction with the proposed method. In this figure, we fitted first one and half cycles to predict the following cycles. Panels (a), (b), and (c) correspond to examples of Type (i), Type (ii), and Type (iii) patients, respectively. In each panel, the blue solid line shows the fitting and the prediction under IAS, the blue vertical dash-dotted line shows the point switching between the fitting and the prediction, and the green dashed line shows the simulation under CAS, and the red crosses show the actually observed values of PSA levels.

Then, we shortened the lengths of PSA time series for the estimation of the prior distribution and used only the first one and half cycles of IAS, meaning the first on- and off- treatment periods and the second on-treatment period. Examples of fitting and prediction using the proposed method are shown in Fig 2. Even when we limited the observation to the first one and half cycles, the subsequent PSA values were predicted well as similarly as Fig. 5 of Ref. [15]. Under the proposed method, we compared the classifications obtained using the first one and half cycles with those obtained using the whole data set (Table 4). We found that these two groups of the classifications matched well. We also compared the classifications by fitting the first one and half cycles with the classifications by the medical doctors (Table 5). We found that their correlation persisted because the p-value was as small as 0.005. In addition, the classifications obtained by the proposed method using the first one and half cycles are consistent with the classifications obtained by the method of Ref. [15] using the whole dataset (Table 6) because in more than the half of patients, the type obtained by the proposed method using the first one and half cycles agreed with the type obtained by the method of Ref. [15] using the whole data. These results mean that the first one and half cycles can provide sufficient information for fitting the datasets for the mathematical model with the proposed method.

**Table 4:** Comparison between the classifications obtained by the proposed method with the whole data (MMWD) and those obtained by the proposed method with the first one and half cycles (MM1H) (the p-value < 0.001).

	MM1H			
MMWD	Type(i)	Type(ii)	Type(iii)	Total
Type (i)	66	17	0	83
Type (ii)	11	28	3	42
Type (iii)	2	2	12	16
Total	79	47	15	141

**Table 5:** Comparison between the classifications by medical doctors (MD) and those by the proposed method with the first one and half cycles (MM1H) (the p-value: 0.005).

	MM1H			
MD	Type(i)	Type(ii)	Type(iii)	Total
Without relapse	56	18	7	81
Metastasis	7	11	3	21
Androgen independence	15	17	5	37
Total	78	46	15	139

**Table 6:** Comparison between the classifications obtained by the proposed method with the first one and half cycles (MM1H) and the classifications by mathematical models using the fitting method of Ref. [15] (MM[15]) (p-value: 0.029).

	MM [15]			
MM1H	Type (i)	Type (ii)	Type (iii)	Total
Type (i)	37	38	4	79
Type (ii)	11	32	4	47
Type (iii)	5	7	3	15
Total	53	77	11	141

**Fig 3:**
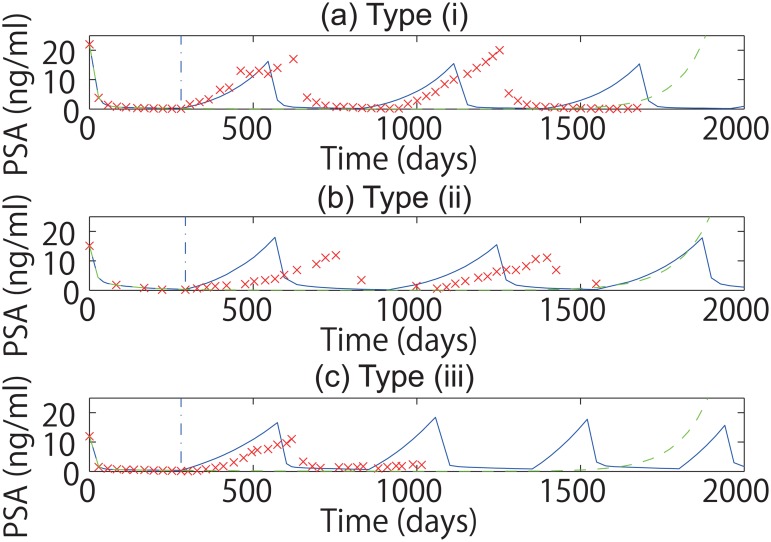
Fitting and prediction using the proposed method. In this figure, we fitted the first half cycle of IAS. To interpret the figure, please see the caption of Fig 2.

However, when we only used the first half cycle of IAS using the proposed method (meaning the first 9–12 months of androgen suppression), we could not obtain the combined parameter values of the mathematical model in such a way that the two groups of the classifications are statistically correlated (see Fig 3, and Tables 7 and 8). In particular, the correlation between the classifications by the mathematical model and the classifications by the medical doctors was lost (Table 8).

**Table 7:** Comparison between the classifications obtained by the proposed method with the whole data (MMWD) and those obtained by the proposed method with the first half cycles (MMH) (the p-value: 0.10).

	MMH			
MMWD	Type(i)	Type(ii)	Type(iii)	Total
Type (i)	59	22	2	83
Type (ii)	23	18	1	42
Type (iii)	13	2	1	16
Total	95	42	4	141

**Table 8:** Comparison between the classifications by medical doctors (MD) and the classifications of mathematical models by fitting the first half cycles (MMH) only using the proposed method (the p-value: 0.80).

	MMH			
MD	Type(i)	Type(ii)	Type(iii)	Total
Without relapse	56	23	2	81
Metastasis	15	5	1	21
Androgen independence	23	13	1	37
Total	94	41	4	139

We tested whether the period before starting the first cycle of the on-treatment period helped to estimate the combined parameters or not. We tested this hypothesis by generating artificial data simulating the off-treatment period of 16 weeks followed by the first half cycles of IAS for the second half of Canadian patients. However, the classifications obtained from the artificial data did not match the classifications of the combined parameter values with which the artificial data were generated (Table 9).

**Table 9:** Comparison between the classifications used for generating the artificial data (MM) and those obtained by fitting the artificial data for the mathematical model using the proposed method (MMAD). The artificial data were generated by simulating the treatment schedule of the off-treatment period of 16 weeks followed by the first half cycle of IAS (the p-value: 0.18).

	MMAD			
MM	Type (i)	Type (ii)	Type (iii)	Total
Type (i)	13	6	0	19
Type (ii)	6	6	2	14
Type (iii)	3	0	0	3
Total	22	12	2	36

## Discussion

There are some correlations between pairs of the combined parameters obtained by fitting time courses of the tumor marker for the mathematical model. By enforcing the prior distribution of the combined parameters constructed by the whole datasets of the past other patients, we can eventually shrink the space of the combined parameters to the space where the combined parameters for the past other patients are distributed. This shrinkage is why the proposed framework enables us to obtain the optimal set of the combined parameters from a short PSA time course of the patient who is currently being treated.

We tested whether or not the distribution of combined parameters $\vec{p}$ follows a multivariate Gaussian distribution by using the skewness and the kurtosis proposed by Mardia [34]. We found that neither the skewness nor the kurtosis was consistent with a multivariate Gaussian distribution; both p-values were less than 0.001 using the *χ* ^2^ distribution and the normal distribution, respectively. This finding implies that we may be able to estimate a set of combined parameters $\vec{p}$ better if we use a more sophisticated distribution such as a mixture of Gaussians [24]. We will follow this direction of research in the future.

There are two possible reasons why Tables 7 and 8 were not statistically significant: The first possible reason is that the first on-period and/or the first on-period with the period before starting the hormone therapy were too short to identify the types of patients; the second possible reason is that the number of patients used for this study was too small to conclude the usefulness for the first on-period and/or the first on-period with the period before starting the hormone therapy. We will answer this point in a future study by analyzing a larger dataset from a phase III trial [26].

The problem considered in this paper, namely estimating the combined parameters for the patient who is currently being treated using information of prior patients, is one of the typical problems we encounter in the context of mathematical medicine. We have another but complementary paper [35] on this topic, within which we extended a method of machine learning so that we can find some patients whose cancer’s temporal behavior was similar to that of a current patient, and take weighted averages for the orbits of the past patients to predict when an additional treatment option is necessary for the current patient. Other problems include finding a treatment schedule such that some medical index is kept within a certain range for a finite time [36–38]; integrating different kinds of information for better diagnosis, treatment, and intervention; and improving the accuracy of a mathematical model while keeping its complexity. Because these mathematical problems have become clearer, it is expected that, in the future, mathematicians will play a significant role in providing efficient personalized therapy based on an individual patient’s data.

If we limit our program for choosing and/or optimizing a treatment option for the hormone therapy of prostate cancer, we now have the proposed method for estimating the initial conditions and parameters as well as some methods for optimizing the treatment schedule [36–38]. Therefore, we will be ready for a clinical trial that tests the validity for the mathematical optimization of the treatment schedule after we clarify whether the first on-period with the off-period beforehand is useful or the first one and half cycles of IAS is necessary for estimating the initial conditions and parameters. This remaining problem is exactly the problem discussed at the second last paragraph.

In sum, we have proposed a method for fitting a relatively short time course of bio-marker levels for a mathematical model of disease when the sets of the Initial conditions and parameters for past other patients are given. We have constructed the prior distribution from the past other patient’s information and formulated a new method of time course fitting by using the Bayesian formula. In this report, we showed that we can estimate a set of the Initial conditions and parameters for the mathematical model of prostate cancer treated with intermittent androgen suppression by using only its first one and half cycles (approximately 2 years). These results are promising and are representative of the potential role of mathematical modeling and rapid learning in the context of decision making in medicine.

## Supporting Information

S1 FileA zipped file containing a program in C named S1_File.c for estimating the set of combined parameters $\vec{p}$, or initial conditions $\vec{x}(0)$ and parameters $d_{i,j}^{m}$ from the dataset of the first one and half cycles of each patient using the prior distribution obtained from the set of the past patients.The input file should be the CSV file format as in ones at http://www.nicholasbruchovsky.com/clinicalResaerch.html, where the columns should be, from the left, the patient number (not used), the date (not used), the amount of prescribed CPA (not used), the amount of prescribed LEU (not used), the PSA level, the testosterone level (not used), the number of cycle, whether the hormone therapy is on (1) or off (0), the accumulated number of days, and the accumulated number of days starting from a different day (not used). Each line corresponds to a day where either the PSA and/or testosterone level was measured or the treatment option was changed. The output is generated in a file called fitted_params8a2c2b2.dat. The output is a single column containing the estimated combined parameters $\vec{p}$. The values from the first line show *x*_1_(0), *x*_2_(0), *x*_3_(0), $d_{1,1}^{1}$, $d_{2,1}^{1}$, $d_{2,2}^{1}$, $d_{3,1}^{1}$, $d_{3,2}^{1}$, $d_{3,3}^{1}$, $d_{1,1}^{0}$, $d_{1,2}^{0}$, $d_{2,2}^{0}$, and $d_{3,3}^{0}$. In the last line, the program outputs the value for the cost function. In between, you find eight 0s, which may be used for the future developments.(ZIP)

## References

[1] Rvachev LA, Longini IM Jr.. A mathematical model for the global spread of influenza. Math Biosci 1985;75: 3–23.

[2] Komarova SV, Smith RJ, Dixon SJ, Sims SM, Wahl LM. Mathematical model predicts a critical role for osteoclast autocrine regulation in the control of bone remodeling. Bone 2003;33: 206–215. 10.1016/S8756-3282(03)00157-1 14499354

[3] Jackson TL. A mathematical investigation of the multiple pathways to recurrent prostate cancer: comparison with experimental data. Neoplasia 2004;6: 679–704. 10.1593/neo.04259 PMC1531673 15720795

[4] Jackson TL. A mathematical model of prostate tumor growth and androgen-independent relapse. Discrete Cont Dyn Syst-Ser B 2004;4: 187–201. 10.3934/dcdsb.2004.4.187

[5] Goldstein ST, Zhou F, Hadler SC, Bell BP, Mast EE, Margolis HS. A mathematical model to estimate global hepatitis B disease burden and vaccination impact. Int J Epidemiol 2005;34: 1329–1339. 10.1093/ije/dyi206 16249217

[6] Anderson ARA. A hybrid mathematical model of solid tumour invasion: the importance of cell adhesion. Math Med Biol 2005;22: 163–186. 10.1093/imammb/dqi005 15781426

[7] Chitnis N, Cushing JM, Hyman JM. Bifurcation analysis of a mathematical model for malaria transmission. SIAM J Appl Math 2006;67: 24–45. 10.1137/050638941

[8] Jain RK, Tong RT, Munn LL. Effect of vascular normalization, by antiangiogenic therapy on interstitial hypertension, peritumor edema, and lymphatic metastasis: insights from a mathematical model. Cancer Res 2007;67: 2729–2735. 10.1158/0008-5472.CAN-06-4102 17363594 PMC3022341

[9] Ideta AM, Tanaka G, Takeuchi T, Aihara K. A mathematical model of intermittent androgen suppression for prostate cancer. J Nonlinear Sci 2008;18: 593–614. 10.1007/s00332-008-9031-0

[10] Shimada T, Aihara K. A nonlinear model with competition between prostate tumor cells and its application to intermittent androgen suppression therapy of prostate cancer. Math Biosci 2008;214: 134–139. 10.1016/j.mbs.2008.03.001 18420226

[11] Guo Q, Tao Y, Aihara K. Mathematical modeling of prostate tumor growth under intermittent androgen suppression with partial differential equations. Int J Bifurcat Chaos 2008;18: 3789–3797. 10.1142/S0218127408022743

[12] Granich RM, Gilks CF, Dye C, De Cock KM, Williams BG. Universal voluntary HIV testing with immediate antiretroviral therapy as a strategy for elimination of HIV transmission: a mathematical model. Lancet 2009;373: 48–57. 10.1016/S0140-6736(08)61697-9 19038438

[13] Tao Y, Guo Q, Aihara K. A model at the macroscopic scale of prostate tumor growth under intermittent androgen suppression. Math Models Meth Appl Sci 2009;19: 2177–2201. 10.1142/S021820250900408X

[14] Tao Y, Guo Q, Aihara K. A mathematical model of prostate tumor growth under hormone therapy with mutation inhibitor. J Nonlinear Sci 2010;20: 219–240. 10.1007/s00332-009-9056-z

[15] Hirata Y, Bruchovsky N, Aihara K. Development of a mathematical model that predicts the outcome of hormone therapy for prostate cancer. J Theor Biol 2010;264: 517–527. 10.1016/j.jtbi.2010.02.027 20176032

[16] Tanaka G, Hirata Y, Goldenberg SL, Bruchovsky N, Aihara K. Mathematical modelling of prostate cancer growth and its application to hormone therapy. Phil Trans R Soc A 2010;368: 5029–5044. 10.1098/rsta.2010.0221 20921010

[17] Kronik N, Kogan Y, Elishmereni M, Halevi-Tobia K, Vuk-Pavlović S, Agur Z. Predicting outcomes of prostate cancer immunotherapy by personalized mathematical models. PLoS ONE 2010;5: e15482 10.1371/journal.pone.0015482 21151630 PMC2999571

[18] Jain HV, Clinton SK, Bhinder A, Friedman A. Mathematical modeling of prostate cancer progression in response to androgen ablation therapy. Proc Natl Acad Sci USA 2011;108: 19701–19706. 10.1073/pnas.1115750108 22106268 PMC3241775

[19] Portz T, Kuang Y, Nagy JD. A clinical data validated mathematical model of prostate cancer growth under intermittent androgen suppression therapy. AIP Adv 2012;2: 011002 10.1063/1.3697848

[20] Morken JD, Packer A, Everett RA, Nagy JD, Kuang Y. Mechanisms of resistance to intermittent androgen deprivation in patients with prostate cancer identified by a novel computational method. Cancer Res 2014;74: 3673–3683. 10.1158/0008-5472.CAN-13-3162 24853547

[21] Akakura K, Bruchovsky N, Goldenberg SL, Rennie PS, Buckley AR, Sullivan LD. Effects of intermittent androgen suppression on androgen-dependent tumors: apoptosis and serum prostate-specific antigen. Cancer 1993;71: 2782–2790. 10.1002/1097-0142(19930501)71:9<2782::AID-CNCR2820710916>3.0.CO;2-Z 7682149

[22] Bruchovsky N, Klotz L, Crook J, Malone S, Ludgate C, Morris WJ, et al Final results of the Canadian prospective phase II trial of intermittent androgen suppression for men in biochemical recurrence after radiotherapy for locally advanced prostate cancer: Clinical parameters. Cancer 2006;107: 389–395. 10.1002/cncr.21989 16783817

[23] Bruchovsky N, Klotz L, Crook J, Goldenberg SL. Locally advanced prostate cancer: Biochemical results from a prospective phase II study of intermittent androgen suppression for men with evidence of prostate-specific antigen recurrence after radiotherapy. Cancer 2007;109: 858–867. 10.1002/cncr.22464 17265527

[24] Bishop CM. Pattern Recognition and Machine Learning. New York; Springer; 2006.

[25] Price KV, Storn RM, Lampinen JA. Differential Evolution: A Practical Approach to Global Optimization. Berlin, Germany: Springer-Verlag; 2005.

[26] Crook JM, OfCallaghan CJ, Duncan G, Dearnaley DP, Higano CS, Horwitz EM, et al Intermittent androgen suppression for rising PSA level after radiotherapy. N Eng J Med 2012;367:895–903. 10.1056/NEJMoa1201546 PMC3521033 22931259

[27] Hussain M, Tangen CM, Berry DL, Higano CS, Crawford ED, Liu G, et al Intermittent versus continuous androgen deprivation in prostate cancer. N Eng J Med 2013;368:1314–1325. 10.1056/NEJMoa1212299 PMC3682658 23550669

[28] Everett RA, Packer AM, Kuang Y. Can mathematical models predict the outcomes of prostate cancer patients undergoing intermittent androgen deprivation therapy? Biophys Rev Lett 2014;9: 173–191. 10.1142/S1793048014300023

[29] Hatano T, Hirata Y, Suzuki H, Aihara K. Comparison between mathematical models of intermittent androgen suppression for prostate cancer. J Theor Biol 2015;366: 33–45. 10.1016/j.jtbi.2014.10.034 25451517

[30] Hirata Y, Akakura K, Higano CS, Bruchovsky N, Aihara K. Quantitative mathematical modeling of PSA dynamics of prostate cancer patients treated with intermittent androgen suppression. J Mol Cell Biol 2012;4: 127–132. 10.1093/jmcb/mjs020 22561841 PMC3612008

[31] Hirata Y, Tanaka G, Bruchovsky N, Aihara K. Mathematically modelling and controlling prostate cancer under intermitent hormone therapy. Asian J Androl 2012;14, 270–277. 10.1038/aja.2011.155 22231293 PMC3735095

[32] Higano C, Shields A, Wood N, Brown J, Tangen C. Bone mineral density in prostate cancer patients without bone metastases who are treated with intermittent androgen suppression. Urology 2004;64: 1182–1186. 10.1016/j.urology.2004.07.019 15596194

[33] Yu EY, Gulati R, Telesca D, Jiang P, Tam S, Russell KJ, et al Duration of first off-treatment interval is prognostic for time to castration resistance and death in men with biochemical relapse of prostate cancer treated on a prospective trial of intermittent androgen deprivation. J Clin Oncol 2010;28: 2668–2673. 10.1200/JCO.2009.25.1330 20421544 PMC2881848

[34] Mardia KV. Measures of multivariate skewness and kurtosis with applications. Biometrika 1970;57: 519–530. 10.1093/biomet/57.3.519

[35] Morino K, Hirata Y, Tomioka R, Kashima H, Yamanishi K, Hayashi N, et al Predicting disease progression from short biomarker series using expert advice algorithm. Sci Rep 2015;5: 8953 10.1038/srep08953 25989741 PMC5386184

[36] Suzuki T, Bruchovsky N, Aihara K. Piecewise affine systems modelling for optimizing hormone therapy of prostate cancer. Philos Trans A Math Phys Eng Sci 2010;368: 5045–5059. 10.1098/rsta.2010.0220 20921011

[37] Hirata Y, di Bernardo M, Bruchovsky N, Aihara K. Hybrid optimal scheduling for intermittent androgen suppression of prostate cancer. Chaos 2010;20: 045125 10.1063/1.3526968 21198137

[38] Hirata Y, Azuma S, Aihara K. Model predictive control for optimally scheduling intermittent androgen suppression of prostate cancer. Methods 2014;67: 278–281. 10.1016/j.ymeth.2014.03.018 24680737

